# Involvement of anesthesiologists in pediatric sedation and analgesia outside the operating room in Japan: is it too late, or is there still time?

**DOI:** 10.1007/s00540-024-03431-4

**Published:** 2024-11-13

**Authors:** Soichiro Obara

**Affiliations:** Teikyo University Graduate School of Public Health, 2-11-1 Kaga, Itabashi-ku, Tokyo 173-8605 Japan

**Keywords:** Procedural sedation and analgesia, Child, Value-based medicine, Workforce

## Abstract

The global COVID-19 pandemic highlighted significant existing supply–demand imbalances in anesthesia workforce, particularly impacting non-operating room anesthesia. Despite documented risks and mortality rates associated with pediatric procedural sedation and analgesia (PPSA) outside the operating room (OR), there is a pressing need for improvements in safety infrastructure. Comparative analysis with international practices reveals that anesthesiologists’ involvement is associated with fewer adverse events and improved outcomes. However, lower reimbursement rate for sedation and anesthesia workforce shortage, and decentralized health resources are contributing factors to limit their participation in PPSA outside the OR in Japan. Enhancing the involvement of anesthesiologists through the public health frameworks such as “high-risk approach” and “population approach” can contribute to improvement of the safety and quality of PPSA. By tackling these challenges and implementing effective solutions, anesthesiologists can play a key role in ensuring safer and more effective PPSA outside the OR. Future challenges include enhancing training, addressing reduced clinical exposure due to work style reform, and developing effective educational systems. Research on improved educational approaches and fundamental outcome indices is crucial for improving PPSA practices outside the OR.

## Introduction

The COVID-19 pandemic highlighted a significant imbalance in anesthesia workforce, particularly in non-operating room (NOR) anesthesia which had been around for long [1, 2]. Many hospitals have altered traditional anesthesiology roles to manage COVID-19 patients [3], which further limited anesthesiologists' availability in NOR settings. For example, a study in Israel found that 75% of departments canceled NOR anesthesia cases during the pandemic [4]. Two years post-pandemic, the anesthesia workforce shortage in U.S. facilities surged to 78% [2], exacerbating existing supply–demand issues, especially in NOR settings [1]. In Japan, recent work-style reform, which aims at improving work-life balance, have imposed restrictions on medical personnel's working hours [5], compounding existing the supply–demand challenges in the anesthesia workforce. These changes may complicate anesthesiologists' involvement in pediatric procedural sedation and analgesia (PPSA) outside the operating room (OR). This manuscript reviews these challenges in Japan and explores potential solutions.

## Underlying issues

### Incidence and risks

The risks of PPSA outside the OR are well-documented in North America, with cardiac arrest occurring in approximately 0.3–0.4 per 10,000 cases [6]. Despite advances in pediatric anesthesia have reduced mortality to around one per 10,000 [7], PPSA outside the OR still contributes significantly to mortality. This highlights the need for safety protocols comparable to those used during general anesthesia in the OR.

### Findings from malpractice analyses

In the U.S., malpractice analyses suggest that early intervention and improved monitoring could reduce severe adverse events (AEs) during procedural sedation and analgesia (PSA) outside the OR [8]. In Japan, investigations into PPSA-related AEs began in 2010, revealing significant issues, especially during pediatric magnetic resonance imaging (MRI) examinations [9]. Despite the introduction of sedation guidelines for pediatric MRI in 2013 [10], approximately 25% of facilities report serious AEs, including respiratory and cardiac arrests [11]. These findings highlight the need for enhanced resources and safety measures in PPSA outside the OR.

### Failure rates of PPSA by non-anesthesiologists outside the OR

Sedation-success rates for imaging procedures performed by non-anesthesiologists vary widely, with failure rates for agents like chloral hydrate, midazolam, and pentobarbital ranging from 2% to 15% [12–14]. For anesthesiologists, accustomed to high success rates in the OR, these figures might seem concerning, although anesthesiologists also encounter failures. A study found failure rates of 13% and 24% for spinal and caudal epidural anesthesia in infants [15]. Anesthesiologists, however, can often switch to general anesthesia, avoiding procedure cancelations. Conversely, failed sedation by non-anesthesiologists can lead to procedure cancelations, causing significant disruptions for families and impacting their work or household responsibilities.

### Barriers to anesthesiologist’s involvement in PPSA outside the OR in Japan

Two primary factors currently limit anesthesiologists' involvement in PPSA outside the OR in Japan. First, the work-style reform has prioritized OR duties, exacerbating the existing anesthesia workforce shortage (total density of anesthesia providers in Japan: 3.69 per 100,000 population) [16]. Second, reimbursement for sedation by anesthesiologists is significantly lower than for general anesthesia under Japan’s national medical fee schedule (sedation: $75 vs. general anesthesia: over $400, based on a conversion rate of 1 Japanese Yen to 0.0068 USD as of August 20, 2024). The lower reimbursement can be a contributing factor to discourage anesthesiologists from participating in PPSA due to minimal facility revenue impact.

## Integrating public health perspectives and anesthesiologist roles

### Historical perspective on anesthesiologists' role in PPSA outside the OR

Recent studies indicate that non-anesthesiologists, such as pediatric intensivists, achieve high success rates as high as 99.3% and serious AE rates as low as 2.7% in PPSA [17]. Despite this, the debate over whether anesthesiologists or non-anesthesiologists should administer PPSA persists [18]. Historically, anesthesiologists’ involvement has led to reduced AEs, higher success rates, and better image quality [19–21].

### Integrating public health perspectives into clinical medicine

Integrating public health perspectives into clinical medicine can enhance both individual and population health by bridging the gap between these disciplines [22]. While public health has historically driven significant population health improvements, collaboration between clinical and public health professionals is crucial for addressing complex health challenges [23, 24]. The COVID-19 pandemic highlighted the benefits of such integration [24].

### High-risk approach perspective

**Fig. 1:**
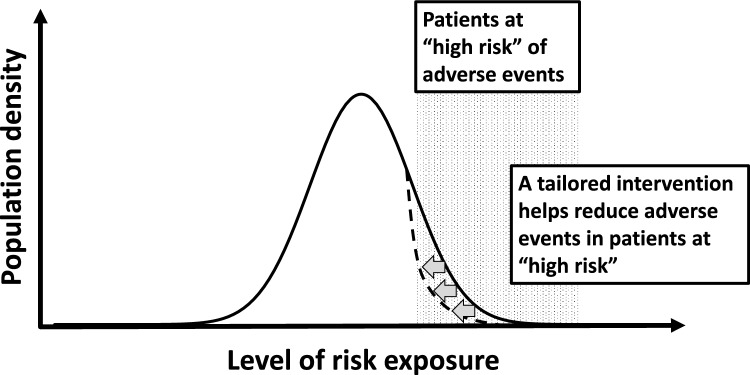
High-risk approach to sedation outside the operating room. This figure illustrates the concept of the “high-risk” approach which aims at minimizing adverse events in patients most susceptible to adverse events during sedation outside the operating room. This approach emphasizes the identification of high-risk individuals—such as those with a history of sedation failure, airway issues, or respiratory and circulatory complications. Following this identification, tailored interventions are implemented to address the specific needs of these patients

From a public health perspective, in what ways could anesthesiologists contribute? One approach is the "high-risk approach" which aims to lower the risk levels of high-risk individuals [25] (Fig. 1). Ideally, anesthesiologists would manage cases with a high likelihood of AEs. A North American study reported severe AEs in 1.78% of cases, with upper airway obstruction being the most common [26]. Key risk factors include obstructive sleep apnea (OSA), developmental delays, and other comorbidities. Additional risk factors for sedation failure include older age, higher ASA-PS class, psychosomatic behavior problems and inflexible temperament, history of sedation failure, upper respiratory tract infections, obesity, congenital syndromes, oxygen dependency, and longer or more stimulating procedures [27–29]. Effective management of these high-risk cases would ideally involve anesthesiologists, necessitating close coordination and information sharing with non-anesthesiologist healthcare providers.

### Population approach perspective

**Fig. 2:**
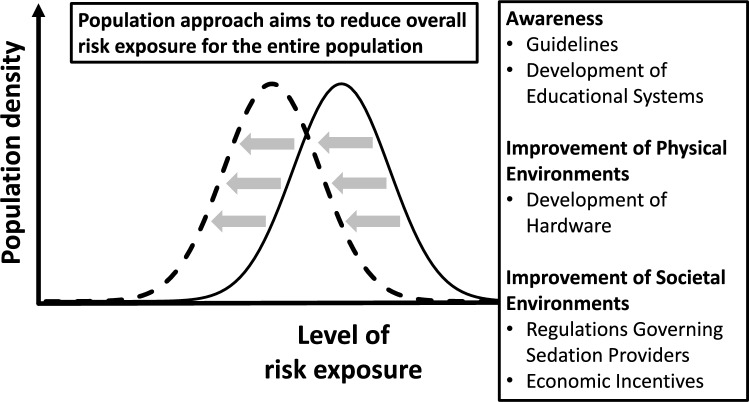
Population approach in sedation outside the operating room. This figure illustrates the "population approach" to managing sedation outside the operating room (OR). This strategy aims to reduce overall risk exposure for the entire population by focusing on three key components: (1) raising awareness through development of comprehensive guidelines and establishment of educational systems; (2) improving physical environments through development of advanced hardware and equipment to enhance safety and efficacy; and (3) improving societal environments through implementation of regulations governing sedation providers and introduction of economic incentives, such as reimbursement, to support safe sedation practices

The high-risk approach has limitations, particularly in disease prevention for those at lower, but non-optimal, risk [30]. In contrast, the "population approach" seeks broad interventions to reduce risk across the entire population (Fig. 2). This approach emphasizes prevention, risk factor modification, and health promotion [31]. In PPSA outside the OR, it includes: (1) raising awareness through development of guidelines and educational programs, (2) improving physical environments through development of advanced equipment, and (3) enhancing societal environments through regulations governing sedation providers and introduction of economic incentives.

In Japan, the Japanese Society of Anesthesiologists published the 2023 "Practical Guide for Safe Sedation" which addresses important aspects but lacks extensive coverage of pediatric cases [32]. Although the Japan Pediatric Society has offered sedation courses, comprehensive educational programs led by anesthesia societies are still needed. In contrast, U.S. anesthesiologists have significantly enhanced PPSA safety through established guidelines and protocols [33]. U.S. anesthesiology residents are required to complete a mandatory rotation in NOR settings [34]. While foreign practices cannot directly be applied due to different healthcare systems, the best practices and innovations can be adapted and incorporated to fit the Japan’s unique context.

## Foundations for improving quality

If anesthesiologists in Japan were to engage indirectly in PPSA outside the OR, how could they contribute to improving "quality"? Quality can be defined by the successful and safe completion of procedures or examinations. To reduce failure rates and prevent complications, key considerations include: (1) clarifying sedation and analgesia goals, (2) assessing sedation levels, (3) understanding and mitigating risk factors for AEs, and (4) comprehending pharmacokinetics.

### Clarifying sedation and analgesia goals

Sedation and analgesia goals vary by procedure and patient needs. Simple procedures like X-rays and ultrasounds require minimal sedation, while MRI often necessitate deep sedation, and highly invasive procedures may require general anesthesia. Proper understanding of these needs is essential for effective planning.

### Assessing sedation levels

Various sedation scales have been developed for different age groups and developmental stages. One such scale is the Richmond Agitation-Sedation Scale (RASS), applicable from 2 months to 21 years of age, regardless of intubation status. The RASS involves observational assessments and responses to verbal and physical stimuli. Although its practical application vary, it provides valuable information on sedation depth, such as identifying deep sedation when responses to verbal stimuli are absent.

### Understanding and mitigating risk factors for adverse events

The Pediatric Sedation Research Consortium, which reviewed over 430,000 cases, reported a 1.78% incidence of serious AEs, with airway obstruction being the most common [26]. Tools like the STBUR (Snoring, Trouble Breathing, Un-Refreshed) questionnaire identify patients at risk for obstructive sleep apnea, which triples the risk of perioperative respiratory complications [35]. Early detection of airway obstruction can be enhanced by capnography and oxygen monitoring. To mitigate apnea risk, it is essential to prepare emergency medications, and avoid excessive polypharmacy.

### Understanding pharmacokinetics

Recognizing risks associated with long-acting drugs or multiple sedatives is crucial for minimizing AEs. An epidemiologic study in Japan showed that the incidence of AEs increased with the number of sedatives (≥ 3; adjusted Oodds ratio, 5.10) and in unscheduled settings (adjusted Odds ratio, 6.28) [36].

Understanding drug administration routes and pharmacokinetics is essential. Intravenous administration generally ensures more reliable absorption and quicker onset than intramuscular, subcutaneous, or oral routes. Key pharmacokinetic considerations involve trichloroethanol and chloral hydrate, both of which are prodrugs metabolized in the liver. These agents have long half-lives, particularly in neonates, and have been linked to serious AEs, including fatalities and neurologic complications in U.S. [37, 38]. Despite decreased overall use, these long-acting sedatives are still utilized in about 50% of cases requiring sedation for MRI in Japan [36].

Recently, intranasal dexmedetomidine and intravenous remimazolam have emerged as promising options for pediatric sedation. Intranasal dexmedetomidine does not cause respiratory depression and shows higher success rates than chloral hydrate or midazolam, achieving 93% success in pediatric MRI cases with initial doses of 4 µg/kg and additional dose of 2 µg/kg as needed [39, 40]. However, it has limitations including delayed onset and potential bradycardia and hypotension [41]. In contrast, remimazolam has a favorable pharmacokinetic profile of high clearance and short context-sensitive half-time [42]. Remimazolam at 0.9–1.2 mg/kg/h maintained spontaneous respiration and allowed for rapid recovery in cases of endoscopy or fiberoptic bronchoscopy [43, 44]. However, side effects such as apnea and hypopnea have been reported [45]. Further research is needed to determine its optimal applications and dosing for PPSA.

## Value-based sedation practice

The shift from evidence-based medicine (EBM) to value-based medicine (VBM) reflects an evolution in healthcare. While EBM emphasizes objective evidence, VBM incorporates patient-perceived value and cost-effectiveness [46]. VBM evaluates interventions by combining quality of life improvements with longevity, providing a comprehensive measure of worth [47]. By integrating patient values with the best evidence, VBM helps physicians deliver higher-quality care and estimate clinical outcomes using healthcare economics.

In PPSA, “value” reflects the ratio of outcomes to resources expended, emphasizing the importance of procedural success, safety, and patient satisfaction [48]. A sensitivity analysis revealed that sedation by non-anesthesiologists is less cost-effective as failure rates rise compared to sedation by anesthesiologists in the current Japanese national medical fee schedules [48]. Higher failure rates in non-anesthesiologist sedation and low reimbursement for sedation suggest that anesthesiologist-administered sedation would be more cost-effective.

Interdisciplinary communication is vital for achieving VBM [49]. For example, a multidisciplinary team including cardiologists, surgeons, and anesthesiologist minimizes risks and optimizes treatment for patients with congenital heart disease (CHD) [50]. This collaboration can enhance resource allocation and improve risks. Pediatric cardiac catheterization has been recognized as carrying significant risks. Up to 25% of cardiac arrests in children occur in those with CHD, with 17% occurring in the catheterization lab [51]. A multicenter registry revealed that 41% of arrests involved patients with cardiac disease, and over half were under one year old [52]. The IMPACT registry reported a major adverse event rate of 7.2% in pediatric catheterization [53]. An audit in Japan found that 27% of institutions experienced major complications requiring surgical intervention or resulting in death [54]. Pediatric anesthesiologists have significantly reduced cardiac arrests in infants compared to non-pediatric anesthesiologists [55], though a recent study found no significant difference in AEs between cardiac and non-cardiac anesthesiologists [56]. Some studies indicate a trend toward reducing catheterizations in favor of non-invasive imaging, including justifying pediatric open-heart surgery without diagnostic catheterization based on CHD type [57, 58]. Following the introduction of echocardiography, diagnostic catheterizations significantly decreased [59], and noninvasive CT reduced the need for diagnostic catheterization in selected cases [60, 61]. This trend enhances patient safety, optimizes resource allocation (such as reallocating anesthesiologists from catheterization labs), and aligns healthcare delivery with patient needs. Through interdisciplinary communication, anesthesiologists can drive value-based perioperative care [62] and improve preoperative optimization in pediatric catheterization.

## Future challenges

The relationship between caseload volume and outcomes in pediatric anesthesia is debated. Evidence indicates that higher experience and caseloads correlate with fewer complications [63–65]. It is recommended that providers manage at least 200 cases annually [64], though defining a “magic” number remains complex [66]. Japan’s residency mandate of 25 cases may be insufficient for developing expertise in pediatric care outside the OR [66]. Recent work-style reform may further limit clinical exposure [5], compounded by a shrinking pediatric population and decentralized healthcare resources, which may exacerbate lower caseloads. Centralization is linked to improved patient outcomes and reduced mortality in various clinical settings [67].

To address these challenges innovative training models exclusively designed for PPSA (e.g., focusing on maintaining spontaneous breathing under sedation) are essential to compensate for reduced caseloads [68, 69]. Hybrid courses combining online lectures and simulations could mitigate the impact of declining caseloads in Japan's super-aging society.

Further research is necessary to assess perioperative pediatric morbidity and mortality in PPSA outside the OR. Establishing multi-institutional research networks like the Pediatric Sedation Research Consortium and evaluating the impact of educational initiatives on clinical performance are essential. In addition, exploring public reporting systems and pay-for-performance models could help adjust reimbursement rate for sedation services [70–72].

## Conclusion

To improve PPSA outside the OR in Japan, it is essential to address the challenges related to anesthesia workforce shortage reduced caseloads under current work-style reform, reimbursement, and decentralized resources. Enhancing the involvement of anesthesiologists through a "high-risk approach" or "population approach" can improve safety and quality.
