# Correction: Involvement of anesthesiologists in pediatric sedation and analgesia outside the operating room in Japan: is it too late, or is there still time?

**DOI:** 10.1007/s00540-024-03449-8

**Published:** 2025-01-09

**Authors:** Soichiro Obara

**Affiliations:** Teikyo University Graduate School of Public Health, 2-11-1 Kaga, Itabashi-ku, Tokyo, 173-8605 Japan

**Correction: Journal of Anesthesia** 10.1007/s00540-024-03431-4

In this article, the dosage information of remimazolam in the section “Understanding pharmacokinetics” was incorrect. The sentence was incorrectly given as “Remimazolam at 9–12 mg/kg/h maintained spontaneous respiration and allowed for rapid recovery in cases of endoscopy or fiberoptic bronchoscopy [43, 44].” but should have been “Remimazolam at 0.9–1.2 mg/kg/h maintained spontaneous respiration and allowed for rapid recovery in cases of endoscopy or fiberoptic bronchoscopy [43, 44].”

The original article has been corrected.

